# Supplementary material for: Digestive glycosidases from cannonball jellyfish (Stomolophus sp. 2): identification and temporal-spatial variability
Source: PeerJ. 2023 Dec 19;11:e16417. doi: 10.7717/peerj.16417 (PMC10740595; doi:10.7717/peerj.16417)
Supplement: Supplemental Information 1 — Protein and glycosidases profiles in the gastric pouches of each of the organisms sampled. [file peerj-11-16417-s001.pdf]

Raw data – Electrophoresis gel

Protein profile

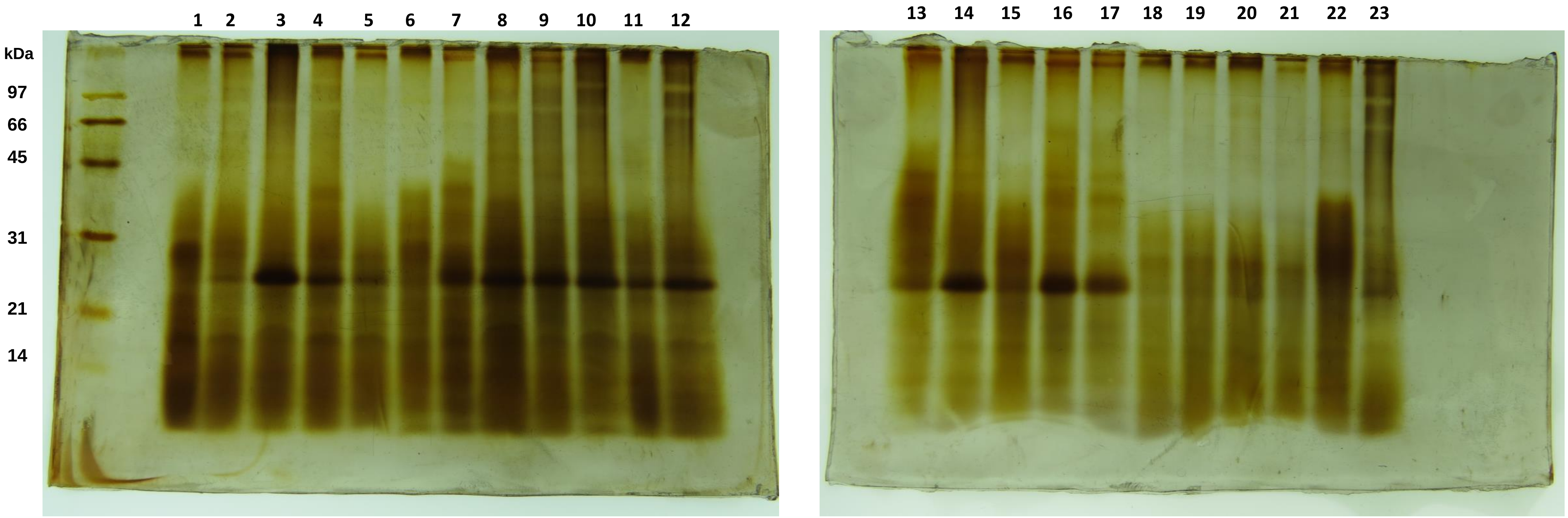

Proteins were separated by electrophoresis, according to Laemmli (1970). A gel of 12% sodium dodecyl sulfate-polyacrylamide (SDS-PAGE) was made to study the protein profile from gastric pouches samples. The gel was loaded with 11 µg of the enzymatic extract from a mixture of the 23 organisms in a 1:1 ratio with a loading buffer (0.125 M Tris-HCl, 4% SDS, 20% v/v glycerol, and 0.02% blue bromophenol). Low molecular weight marker (Bio-Rad; 1610304) was used. Line 1-23: Protein profile of the gastric pouches from each of the 23 organisms sampled; kDa: Kilodalton.

Zymogram of *β*-*N*-acetylhexosaminidase activity

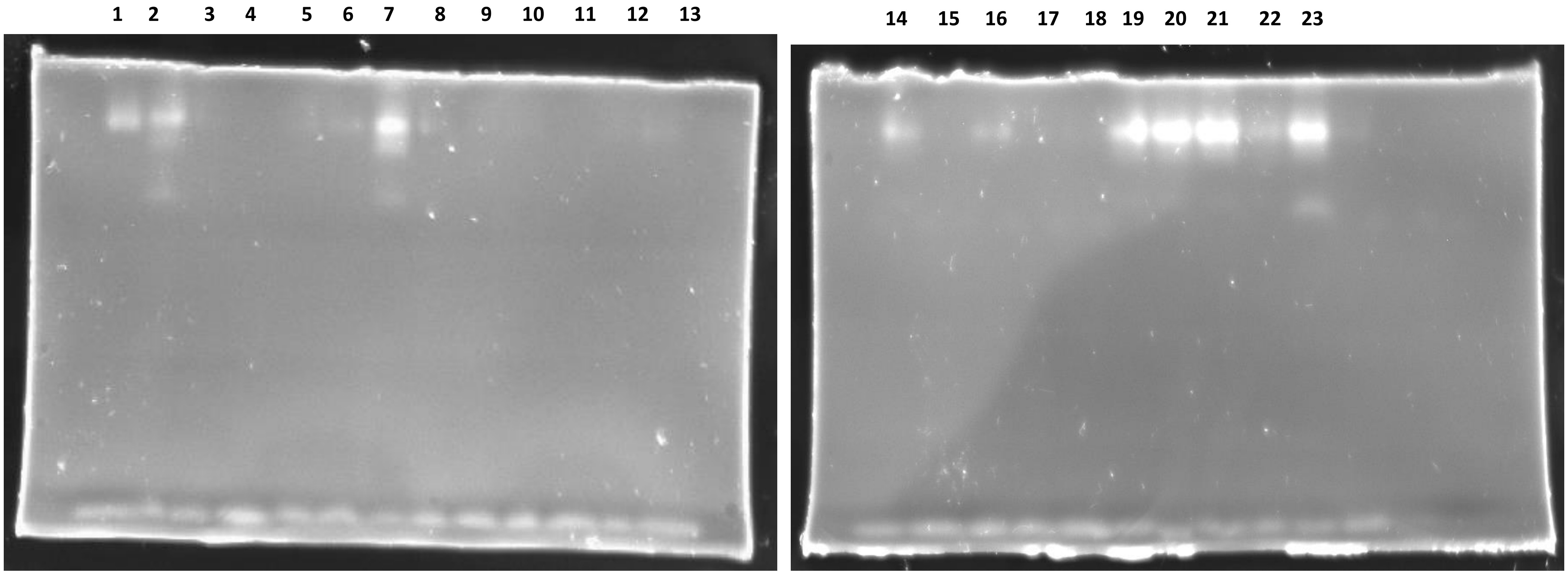

After protein separation, the gel was washed with distilled water and incubated in 30 mL of buffer solution (50 mM Tris-HCl, pH 6.0) for 1 hour. The gel was then transferred to a 100 mM citrate buffer, solution 100 mM pH 5.0 with 2.5% of Triton X-100 2.5% for 60 min, then incubated with 15 mL of fresh citrate buffer a 15 mL solution of with 4-methylumbelliferyl-glucopyranoside (4-MUGLc) (w/v). Lines 1-23: activity profile from each of the 23 organisms sampled.

Zymogram of *β*-glucosidase activity

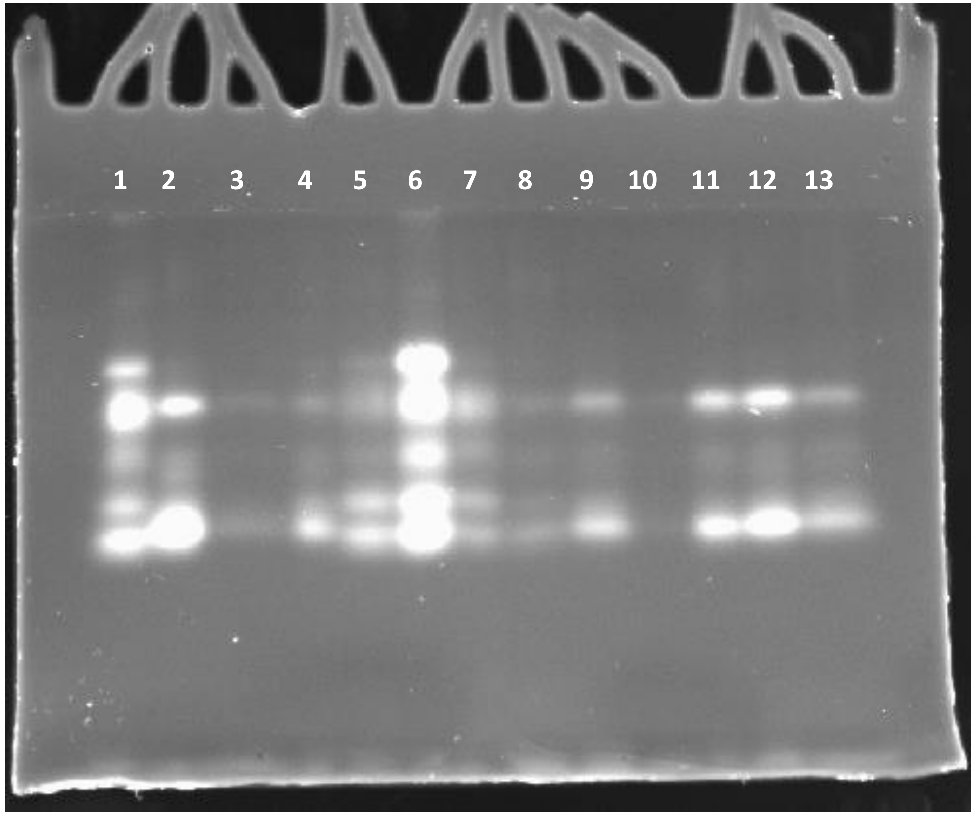

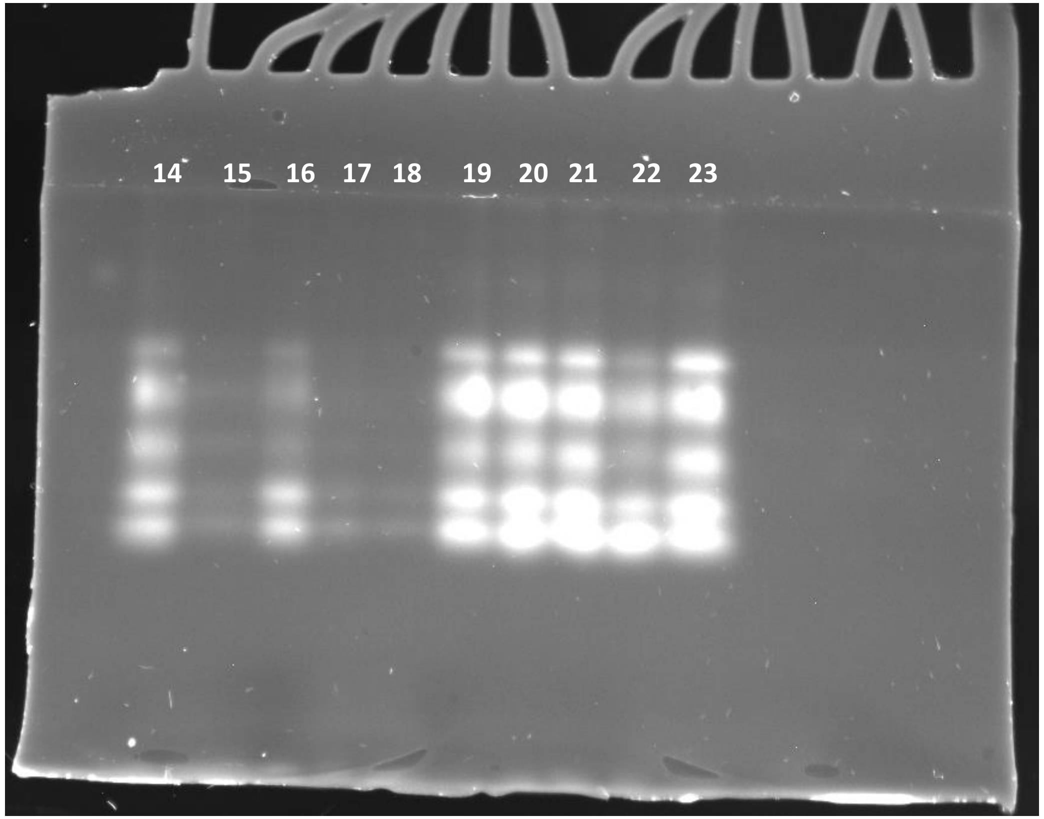

For β-glucosidase, 15 mL of the citrate buffer solution (pH 6.0) with 4-methylumbelliferil cellobioside (4-MUG) (w/v) was used for exoglucanase activity. The incubation was at 37 ° C with agitation (100 rpm) for 10 min, finally visualized with UV light (302 nm). Lines 1-23: activity profile from each of the 23 organisms sampled.

Chitinase activity zymogram

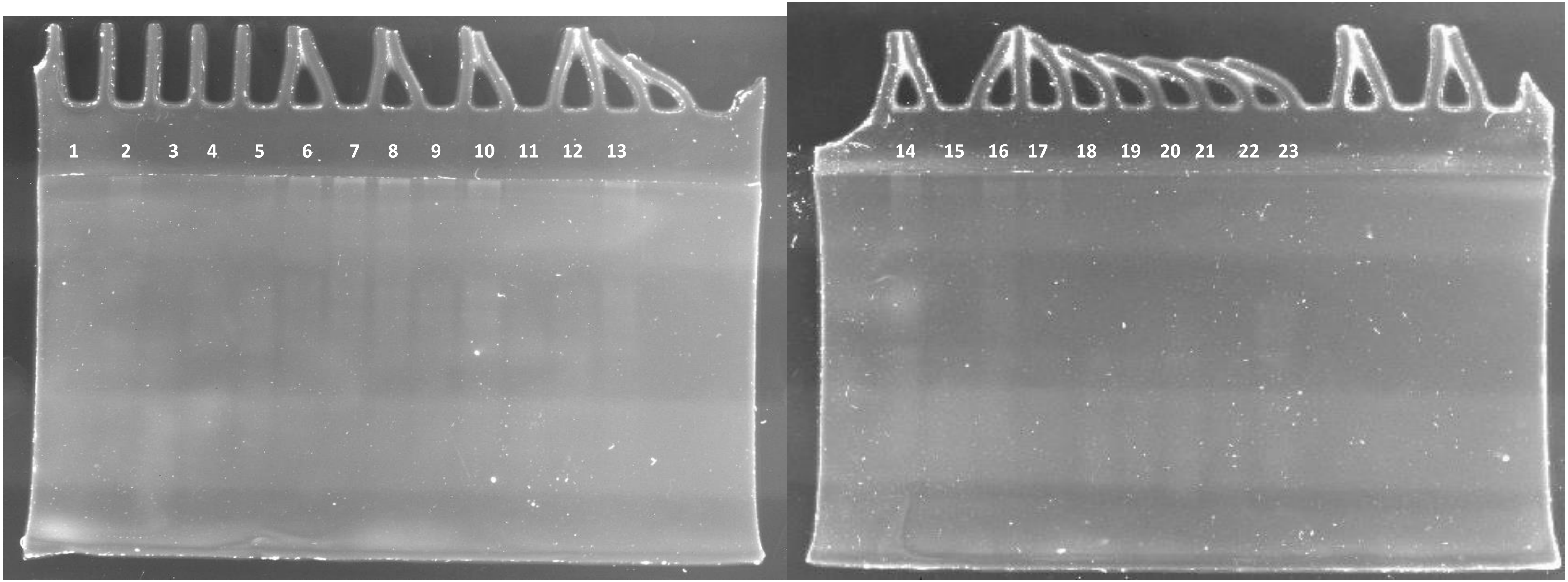

Chitinase activity was identify using 0.01% of glycol chitin copolymerized with the SDS-PAGE, washed according to the abovementioned conditions, then incubated in a solution containing 0.01% Calcofluor White M2R in 0.5 M Tris-HCl pH 8.0, next, incubated for one hour with distilled water at room temperature (25 °C) and photo-documented under UV light (302 nm) (Tronsmo & Harman 1993). Lines 1—23: *β*-*N*-acetylhexosaminidase activity profile from each of the 23 organisms sampled.
