# Supplementary figures and images for: Digestive glycosidases from cannonball jellyfish (Stomolophus sp. 2): identification and temporal-spatial variability
Source: PeerJ. 2023 Dec 19;11:e16417. doi: 10.7717/peerj.16417 (PMC10740595; doi:10.7717/peerj.16417)

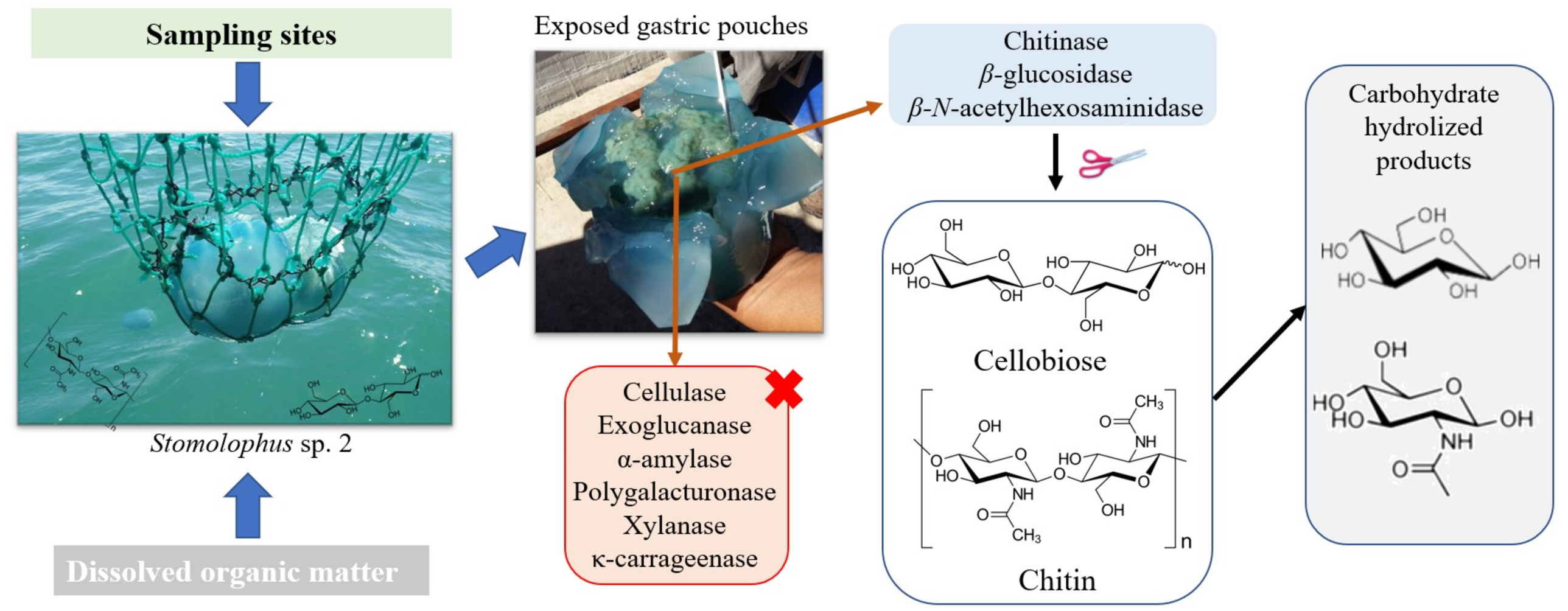

Supplement: Figure S1 — Organisms were cut on top to expose the gastric pouch where the glycosidases are located. [file peerj-11-16417-s003.png]
